# Pollution-Induced Food Safety Problem in China: Trends and Policies

**DOI:** 10.3389/fnut.2021.703832

**Published:** 2021-11-11

**Authors:** Qianhui Li, Kunyang Zhu, Lei Liu, Xinyi Sun

**Affiliations:** School of Public Administration, Sichuan University, Chengdu, China

**Keywords:** environmental pollution, food safety, China, document analysis, policy evolution

## Abstract

Based on systematic literature study and policy document analysis, this paper investigates the environmental pollution-induced food safety problem in China, including the impact of environmental pollution on food safety and the policy response of Chinese government since 1970's. The results show that, to different degrees, food safety of China is affected by large but inefficient chemical fertilizer and pesticides residue (although the consumption began to decline after around 2015), cropland heavy metal pollution (especially cadmium), water pollution, and high ozone concentration. The evolution of pollution-induced food safety policies of China can be divided into four stages, i.e., preparation stage (1974–1994), construction stage (1995–2005), elaboration stage (2006–2013), and intensification stage (2014–). Through the four stages, the increasingly stringent policy system has been featured by “from supply-safety balance to safety first,” “from multi-agency management to integrated management,” and “from *ex post* supervision to *ex ante* risk control.” To further prevent pollution and control food quality, more collaborations between the agricultural and environmental agencies and more specific policies should be anticipated.

## Introduction

Food safety is the basic requirement for human health and public safety. It is both necessary and challenging to ensure food safety as it is related to the whole supply chain, involving production, processing, storage, transport, and trade. In recent decades, along with the transformation from traditional agriculture to intensive farming and the rapid industrialization and urbanization around the globe, environmental pollution has become an important source of food insecurity. The pollution, along with the food production, including soil and water pollution and excessive use of chemical fertilizers and pesticides, will accumulate toxic substances in the agricultural product, such as sulfates, nitrates, and heavy metals (1, 2), which finally threatens human health through the food consumption. Therefore, in areas with relatively poor environmental quality, people are more likely to ingest polluted food and get food-borne diseases (3).

So far, pollution-induced food safety problems have been widely reported around the world. According to the Food and Agriculture Organization (FAO), one third of the plastic produced globally ends up in the soil with plastic particles then entering the food chain and the environment.^1^ By 2050, the ecosystem degradation and climate change could reduce crop yields by 10% globally and by up to 50% in certain regions.^2^ The United Nations Economic Commission for Europe (UNECE) estimated that ozone (O_3_) pollution caused relative global crop losses for soybean, 6–16%; wheat, 7–12%; and corn, 3–5%^3^. Tai et al. (4) estimated that warming would reduce global crop production by over 10% by 2050 with a potential to substantially worsen global malnutrition. At the country level, for example, nearly 60% of the top agricultural soil in 11 European countries has been found to contain residues of multiple persistent pesticides.^3^ In Japan, Harada (5) estimated that, during 1958–1995, 1,043 people died from long-term consumption of seafood containing mercury in Minamata City. In Ghana, the content of cadmium (Cd) and lead (Pb) in the vegetables cultivated in the waste dumpsites of Kumasi was found to be far exceeding the WHO/FAO recommended guideline value (6). In Bangladesh, the arsenic (As) and Cd contained in the rice and vegetables from Brahmanbaria were higher than the established safety limits (7).

Facing with the largest population and food shortage challenge of the world, since 1950's, increasing inputs of inorganic fertilizers, organic manures, and pesticides has become the principal means for China to attain high-crop yields and greater livestock production (8). However, the utilization efficiency of the agricultural input has been relatively low. According to the Ministry of Agriculture of China, as of 2015, the utilization rate of fertilizers and pesticides, the recovery rate of plastic film, and the effective treatment rate of livestock waste were <1/3, 2/3, and 1/2, respectively (9). The high input and low efficiency have contributed substantially to the emissions of greenhouse gases such as CH_4_ and N_2_O, and the entry of pollutants into water bodies and soils, such as nitrogen and phosphorus, pesticide, and heavy metals, which would finally be transferred and accumulated in food. According to the “Circular of the State Administration of Market Regulation on the Food Safety Supervision and Sampling Inspection in the First Half of 2020,” 36.42% of the unqualified food samples contained excessive residues of pesticides and veterinary drugs. In addition, the rapid industrialization and urbanization since the opening and reform at the end of 1970's have further aggravated soil pollution of China. According to the Ministry of Environmental Protection and Ministry of Land Resources (10), about 19.4% of the arable land of the country is polluted. Zhang et al. (11) estimated that about 10.18% arable land of China is polluted by heavy metals, and 13.86% of grain production is thus contaminated.

In order to combat the food security threat induced by industrial pollution and overuse of toxic agricultural inputs, the Chinese government has adopted a series of pointed policies. As early as in 1974, the “Key Points and Main Measures of Environmental Protection Planning” issued by the Environmental Protection Leading Group of the State Council prescribed that “the use of highly residual and highly toxic pesticides in the production of tea, tobacco, Chinese herbal medicine, melon, fruit, vegetables, and other crops should be immediately stopped.” In 1995, China issued the first law on food safety, “Food Hygiene Law of China.” In 2009, the law was replaced by the “Food Safety Law of China,” which “prohibited the production and trade of food, food additives, and food-related products containing pollutants such as pesticide residues, veterinary medicine residues, biotoxins, and heavy metals, and other hazardous substances that exceed the food safety standards.” In 2015 and 2018, the law was amended two times. With the deepening of the supervision over the pollution-induced food safety problem, in 2015, the Ministry of Agriculture and Rural Affairs of China promulgated the “Action Plan for Zero Growth of Fertilizer Use by 2020” and “Action Plan for Zero Growth of Pesticide Use by 2020,” promoting the strictest ever prohibition on the use of agricultural inputs.

With the rising demand for both safe food and a cleaner environment, it is significant to alleviate the pollution-induced food safety problem in China. Although many relevant studies have paid attention to the issue, most of them scatter in different research fields and at different scales, and largely ignore the relevant public policies. Differently, this paper presents a systematic review for the pollution-induced food safety problem in China, including the impact of environmental pollution on food quality and the policy response of the government, which is expected to advance the understanding of the environment-food nexus and shed some light on the targeted policy making.

## The Impact of Pollution on Food Safety in China

The usage of chemical inputs, cropland heavy pollution, sewage irrigation, and air pollution are all the sources of pollution affecting food safety. The overuse of chemical fertilizers and pesticides, the major source of persistent organic pollutants, and endocrine disruptors will impair plant metabolism and pollute the crops. The excessive chemical inputs will also enter soil, water, and air, and aggravate the environmental pollution due to the industrialization and urbanization. Once contacting with the contaminated soil and water, the plants will take up the toxic chemicals, which will be transferred to seeds and edible parts, and finally enter the human body through the food chain.

### Chemical Fertilizer and Pesticide Consumption

**Figure 1:**
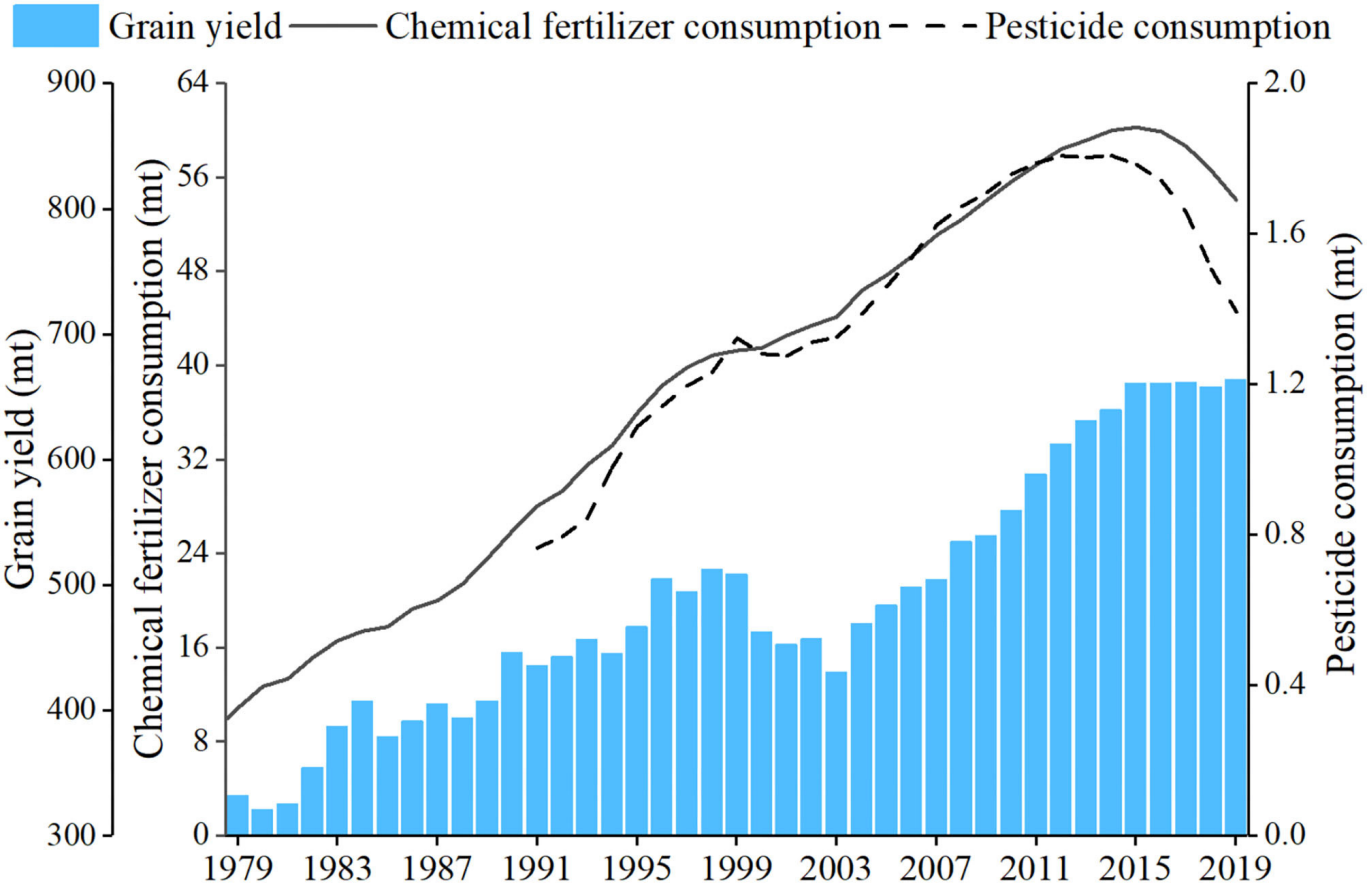
The grain yield and fertilizer and pesticide consumption. Data source: (12).

As shown in Figure 1, with the growth of the grain yield, the chemical fertilizer and pesticide consumption in China kept rising until around 2015. From then on, the consumption has been declining but, so far, is still the largest in the world. In 2019, China consumed 54.03 million tons (mt) of chemical fertilizers, including 19.3 mt of nitrogen fertilizer, 6.81 mt of phosphorus fertilizer, and 5.61 mt of potassium fertilizer (12), which was 30.32% higher than that in the year 2000, and almost two times as much as that of the United States, Japan, and the United Kingdom combined. As of 2019, pesticide consumption in China reached 1.39 mt, an increase of 8% compared with that in the year 2000 (12). Nonetheless, the utilization efficiency of the chemical fertilizer and pesticide in China is relatively low compared with that in developed countries. According to Zhang et al. (13), the average absorption rates of chemical fertilizer and pesticides of rice, corn, and wheat in China are 39.2 and 39.8%, respectively, while the nitrate and pesticides use efficiency in developed countries are 70–80 and 50–60%, respectively.

**Figure 2:**
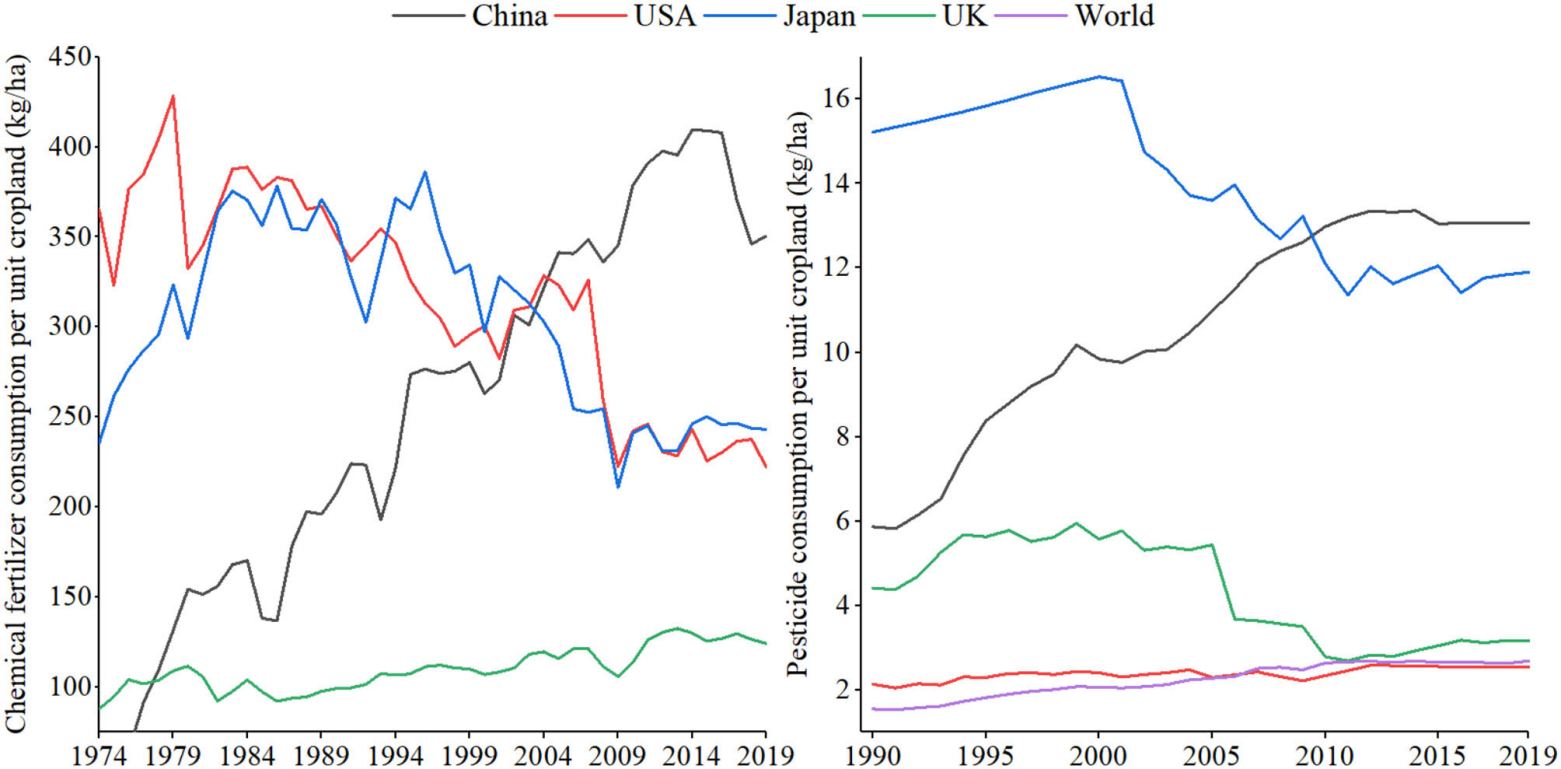
The chemical fertilizer and pesticide consumption intensity in selected countries. Data source: Statistics Division of the Food and Agriculture Organization.

What is worse is that the consumption intensity of chemical fertilizer and pesticide in China has almost kept rising during the past two decades, as shown in Figure 2. As of 2019, the fertilizer consumption intensity of China was 350.5 kg/ha, which was about 1.5 times that of the United Kingdom and Japan, three times that of the United States and world average (122.01 kg/ha).^4^ The pesticide consumption intensity of China in 2019 was 13.07 kg/ha, which was about four times that of the United Kingdom, five times that of the United States and global average (2.69 kg/ha)^4^.

The extensive use of chemical inputs may boost the grain yield in short term, but the high residue is a big threat to food quality. In the inspection of the State Administration of Market Regulation in the first half of 2020, 36.42% of the unqualified food samples were still due to excessive residues of agricultural and veterinary drugs.^5^ According to Zeng et al. (14), the cropland with excessive dichlorodiphenyltrichloroethane (DDT) and polynuclear aromatic hydrocarbons (PAH) accounts for 3.75 and 17.02% of the total cropland of China, respectively. According to the investigations in different regions (Table 1), the organophosphate pesticide residues are commonly higher than relevant national standards.

**Table 1:** Organophosphate pesticide residues in grain.

**Location**	**Detection rate (%)**	**Rate over maximum residue limit (%)**	**Sample number**	**Sampling time**	**References**
Guangdong province	9.6	4.0	125	2001	(15)
Henan province	0.0	0.0	1,000	2007	(16)
Yingcheng city, Hubei province	30.0	10.0	60	2007	(17)
Tianmen city, Hubei province	12.0	4.0	25	2008	(18)
Jilin province	25.5	24.5	102	2015	(19)
Beijing	30.0	20.0	22	2016	(20)

Moreover, the non-point agricultural pollution containing chemical fertilizers and pesticides has put much pressure on the quality of soil, surface water, groundwater, and, finally, farm products, constituting a vicious circle between pollution and food production. According to the estimation of Shen et al. (21), the nitrogen surplus from crop production would increase from about 154 kg/ha in 2004 to 179 kg/ha in 2015. Sun et al. (8) also predicted that if current policies and trends continued, Hainan, Anhui, Hebei, and Chongqing would face high risks of non-point agricultural pollution in 2020.

### Cropland Heavy Metal Pollution

The intake of pollutants *via* the soil-crop-food chain is the predominant pathway of human exposure to toxic substances. Among all types of pollutants, heavy metals are considered to present the greatest risk to food safety. Currently, about 10.18% of the cropland of China is polluted by heavy metals, and 13.86% of grain production is thus damaged (11). Long-term exposure to heavy metals can cause serious health hazards, such as diarrhea, abortion, hepatitis, and typhoid.

**Figure 3:**
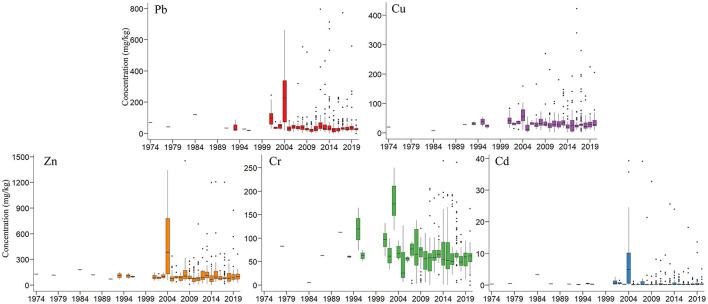
The average heavy metal concentrations in agricultural soil in China (1974–2020). Data source: Data for 1974–2000 are compiled from Zhang et al. (22), Lin (23), Ding et al. (24), Wang (25), Feng et al. (26), Hu et al. (27), Ma and Xu (28), Yan (29), Zhou et al. (30), Liao (31), Wang et al. (32), Wen and Wei (33), and Zhang et al. (34). Data for 2000–2018 are from Zeng et al. (14). Data for 2019–2020 are compiled from Ali et al. (35), Chai et al. (36), Chen et al. (37), Guan et al. (38), Guo et al. (39), Han et al. (40), Hou et al. (41), Hu et al. (42), Huang et al. (43), Jiang and Guo (44), Jin et al. (45), Jin et al. (46), Lu et al. (47), Lv and Sun (48), Song et al. (49), Wang et al. (50), Wu et al. (51), Xiao et al. (52), Yang et al. (53), Zhao et al. (54), Zhou et al. (55), Bao et al. (56), Cao et al. (57), Chai et al. (58), Cheng (59), Duan et al. (60), Guo et al. (61), He et al. (62), Hu et al. (63), Ji et al. (64), Kuerban et al. (65), Li et al. (66), Ma et al. (67), Miao et al. (68), Peng et al. (69), Shi et al. (70), Su and Yang (71), Sun et al. (72), Tan et al. (73), Tang et al. (74), Wei et al. (75), Xiao et al. (76), Zhang et al. (77), Zhang et al. (78), Zhao et al. (79), Zhuang et al. (80).

Through a thorough review and calculation based on the existing studies, Figure 3 shows the heavy metal concentrations in cropland in China during 1974–2020. According to the Spearman coefficient between the concentrations and years, statistically, before 2004, the concentration of Pb (coef. = 0.2367, *p* = 0.0224), Cu (coef. = 0.3621, *p* = 0.0029), Zn (coef. = 0.3897, *p* = 0.0002), and Cd (coef. = 0.3273, *p* = 0.0020) showed a significant increasing trend. After reaching the peak in 2004, the concentration of Pb declined significantly (coef. = −0.0790, *p* = 0.0113). For other metals, the concentrations did not show a statistically significant trend. Before 2003, the concentration of Cr increased significantly (coef. = 0.5559, *p* = 0.0000). After reaching the peak in 2003, the concentration fluctuated without a clear trend.

Overall, from 1974 to 2020, the average concentration of Cu (coef. = −0.0991, *p* = 0.0029), Cr (coef. = −0.1439, *p* = 0.0000), and Pb (coef. = −0.1673, *p* = 0.0000) declined significantly. Currently, according to the national standard in Table 2, except Cd, the heavy metal concentrations are basically at a safe level after 2004.

**Table 2:** Risk control standard for soil contamination of agricultural land (GB 15618-2018).

**Heavy metal**		**Risk screening value (mg/kg)** ^***a***^				**Risk control value (mg/kg)** ^***b***^			
		**PH ≤ 5.5**	**5.5 < PH ≤ 6.5**	**6.5 < PH ≤ 7.5**	**PH > 7.5**	**PH ≤ 5.5**	**5.5 < PH ≤ 6.5**	**6.5 < PH ≤ 7.5**	**PH > 7.5**
Cd	Paddy field	0.3	0.4	0.6	0.8	1.5	2.0	3.0	4.0
	Other	0.3	0.3	0.3	0.6				
Pb	Paddy field	80	100	140	240	400	500	700	1,000
	Other	70	90	120	170				
Cr	Paddy field	250	250	300	350	800	850	1,000	1,300
	Other	150	150	200	250				
Cu	Orchards	150	150	200	200	-	-	-	-
	Other	50	50	100	100				
Zn		200	200	250	300	-	-	-	-

a *If the heavy metal concentration in the soil is larger than the risk screening value, the edible agricultural products may not meet the quality and safety standards*.

b *If the heavy metal concentration is higher than the risk intervention value, the pollution risk of edible agricultural products not meeting the quality and safety standards is high, and strict measures, such as banning the planting of edible agricultural products and returning farmland to forests, should be adopted*.

**Figure 4:**
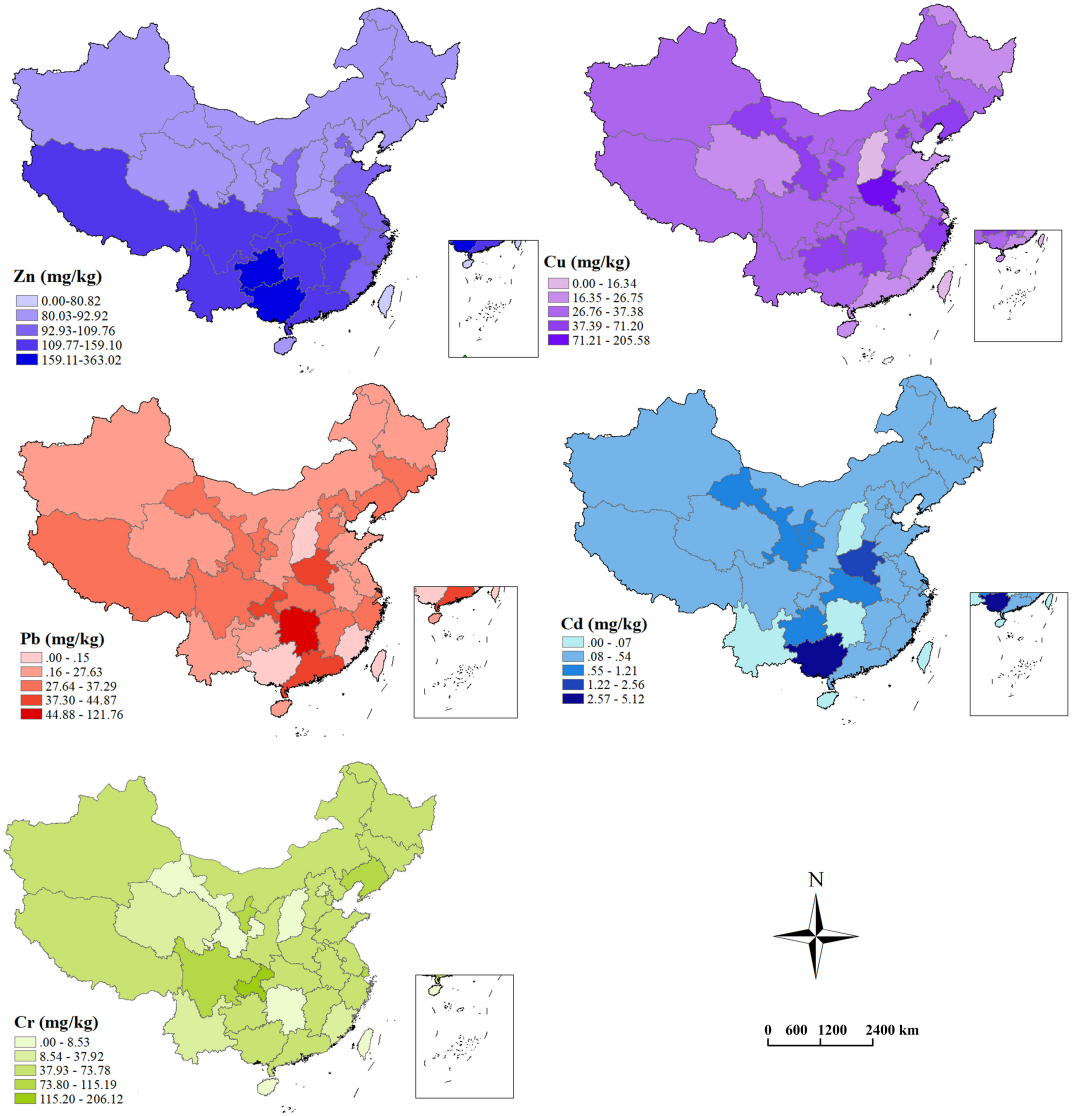
The heavy metal concentrations in the farmland soil in different areas.

Figure 4 shows the heavy metal concentrations in the cropland in different provinces (Macao and Hongkong are excluded due to limited data). Accordingly, the Zn concentration in the Guangxi autonomous region is the largest, reaching 363.02 mg/kg, which is 21% higher than the largest risk screening value in Table 2 (300 mg/kg). Following Guangxi are Guizhou, Hunan, and Sichuan provinces, with the Zn concentration of 173.36, 140.19, and 135.02 mg/kg, respectively. The Cu concentration in Henan province is the largest, reaching 205.58 mg/kg, 2.79% higher than the largest risk screening value in Table 2 (200 mg/kg). Following Henan are Liaoning, Gansu, and Hunan provinces, with the Cu concentration of 68.21, 53.06, and 41.18 mg/kg, respectively. The Pb concentration in Hunan province is the largest (121.76 mg/kg), followed by Chongqing City, Guangdong and Henan provinces, with the Cu concentrations of 44.87, 44.57, and 40.77 mg/kg, respectively. The Cd concentration of Guangxi autonomous region is the largest, reaching 5.12 mg/kg, 28% higher than the largest risk control value (4 mg/kg). The following are Henan, Hubei, and Gansu provinces, with the Cd concentration of 2.56, 1.21, and 1.05 mg/kg, respectively. The area with the largest Cr concentration is Chongqing City (206.12 mg/kg), followed by Sichuan province (115.19 mg/kg), the Ningxia autonomous region (81 mg/kg), and Liaoning province (57.9 mg/kg).

Overall, the cropland heavy metal pollution in the central and southwestern China is relatively serious, represented by the provinces and cities, such as Hunan, Henan, Guangxi, Sichuan, and Chongqing. Gansu and Liaoning provinces in north China are also prominent in heavy metal pollution. Out of the heavy metals, Cd pollution is the most serious in current China.

**Figure 5:**
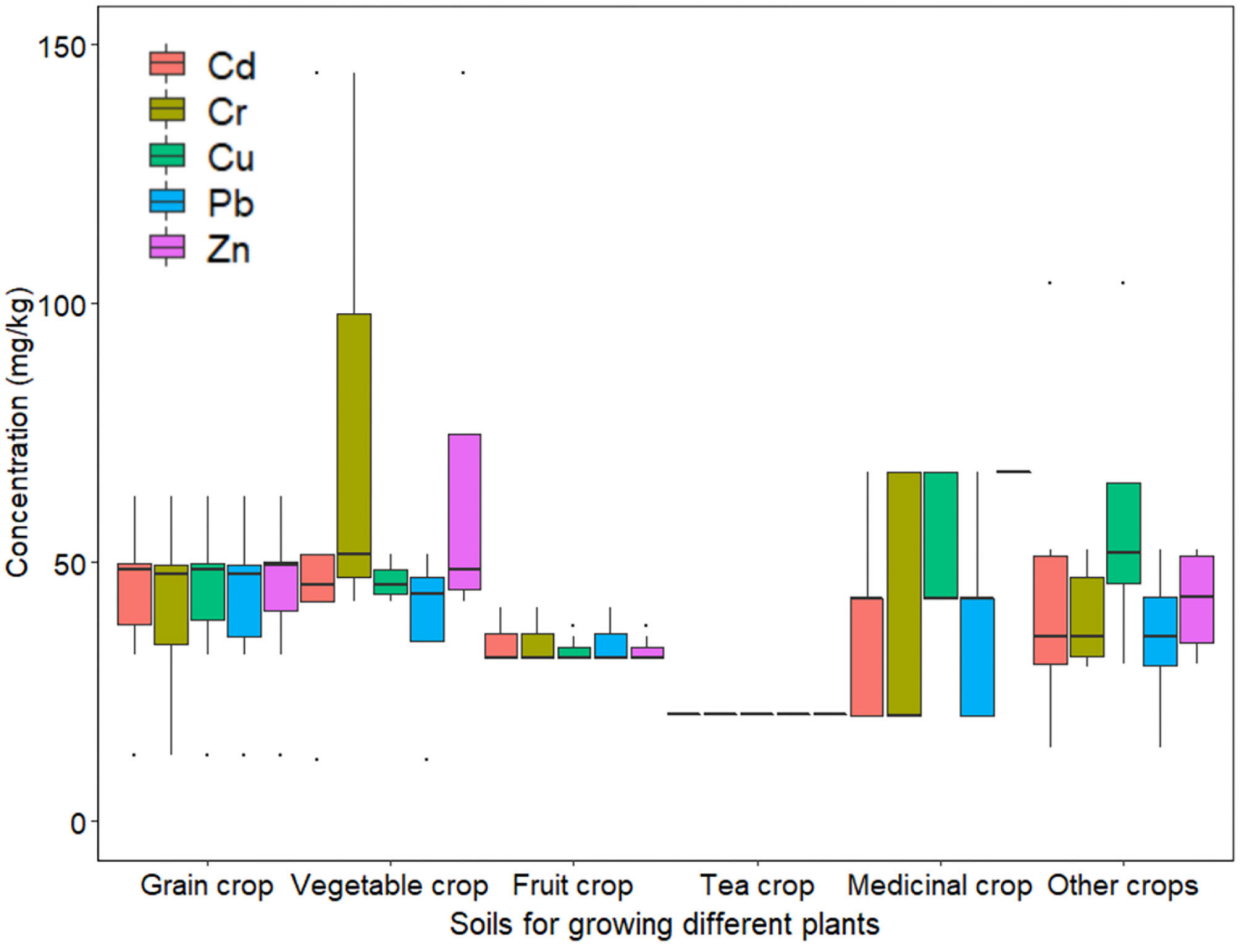
The heavy metal concentration in soil for growing different plants.

Figure 5 summarizes the heavy metal concentrations of different types of agricultural soil in terms of the main crops planted. Accordingly, the heavy metal concentrations in the soil for growing vegetables are relatively high, which is similar to the results of the previous studies (81).

During the past two decades, a series of incidents on the heavy metal pollution in crop has been reported, which has threatened food security of China and even social stability. Table 3 lists 10 typical incidents.

**Table 3:** Ten typical heavy metal contamination events in the past two decades.

**Year**	**Location**	**Event**
2005	Shaoguan city, Guangdong province	The surrounding river and cropland of a village was polluted by the Dabao Mountain Mine. In cropland, Pb concentration reached 225 ppm, 44 times of national standard, and Cd concentration reached 625 ppm, 18 times of national standard. Cd and Pb were also detected to be significantly high in the villagers' diet.
2006	Zhuzhou city, Hunan province	The crops and soil of a village was polluted by the discharge of industrial pollution and high Cd-content fertilizer. Mild cadmium poisoning was found for 150 villagers.
2009	Liuyang city, Hunan province	A village was polluted by a chemical plant producing zinc sulfate. The crops surrounding the plant got disease due to high Cd content. The Cd concentration in the urine of 509 of 2,888 people was diagnosed to exceed standard.
2009	Xi'an city, Shanxi province	The heavy metal content in many places in suburb Xi'an seriously exceeded the environmental standard. The copper in wheat and corn exceeded the standard by 2–5 times, and the Ld exceeded the standard by 2–6 times.
2011	Qujing city, Yuannan province	A chemical plant illegally transferred more than 5,000 tons of chromium slag, leading to the hexavalent Crin the farmland and aquifers reaching 200 and 106 times of standard.
2012	Yizhou district, Guangxi province	Longjiang River was seriously polluted by a mine factory and a flour mill, and the Cd content in the river reached 80 times of the standard.
2013	Dapu county, Hunan province	In a rice sampling and testing, more than half of rice from Dapu county, Hunan province were found to contain excessive Cd. In Youxian county, 90% of paddy soil had Cd contamination and over 70% of the grain samples exceeded the maximum safe concentration.
2014	Daxin county, Guangxi province	The cropland in a village was polluted by the sewage discharged by a lead zinc mine, and the Cd content exceeded the standard by 30 times.
2017	Jiujiang city, Jiangxi province	The Cr content in rice samples from Jiujiang city was found to be 1.56 times of standard.
2017	Xinxiang city, Henan province	The Cd content in all the 12 random wheat samples from Xinyang, collected by an environmental NGO, was found to exceed the standard by 1.7–1.8 times.

*^a^The Shaoguan Smelter water pollution incident happened accidentally? https://www.h2o-china.com/news/43711.html (In Chinese) (accessed September 19, 2021)*.

*^b^The cadmium pollution incident in Liuyang: criminal detention of legal representative and the suspension of environmental protection director. http://www.gov.cn/jrzg/2009-08/02/content_1381586.htm (In Chinese) (accessed September 19, 2021)*.

*^c^CPPCC members warned that many places in the suburbs of Xi'an were polluted by heavy metals. https://xian.qq.com/a/20120112/000061.htm (In Chinese) (accessed September 19, 2021)*.

*^d^The Cd pollution event in Qujing, Yunnan. http://env.people.com.cn/GB/211746/228644/ (In Chinese) (accessed September 19, 2021)*.

*^e^Expert: long-term excessive exposure to cadmium can cause chronic poisoning. http://news.cntv.cn/china/20120129/105818.shtml (In Chinese) (accessed September 19, 2021)*.

*^f^“China's first cadmium rice case” lost, lawyer: continue to fight a “losing” lawsuit. http://www.thepaper.cn/baidu.jsp?contid=1374355 (In Chinese) (accessed September 19, 2021)*.

*^g^A village in Daxin, Guangxi was polluted by heavy metals, and hands and feet of villagers were deformed. http://pic.people.com.cn/n/2014/1208/c1016-26166200.html (In Chinese) (accessed September 19, 2021)*.

*^h^He was shocked by the “cadmium rice” incident in Jiujiang again. https://www.sohu.com/a/204782866_760587 (In Chinese) (accessed September 19, 2021)*.

*^i^Investigation on cadmium pollution of wheat in Xinxiang, Henan, a grain production base. http://m.people.cn/n4/2017/0708/c676-9291738.html (In Chinese) (accessed September 19, 2021)*.

**Figure 6:**
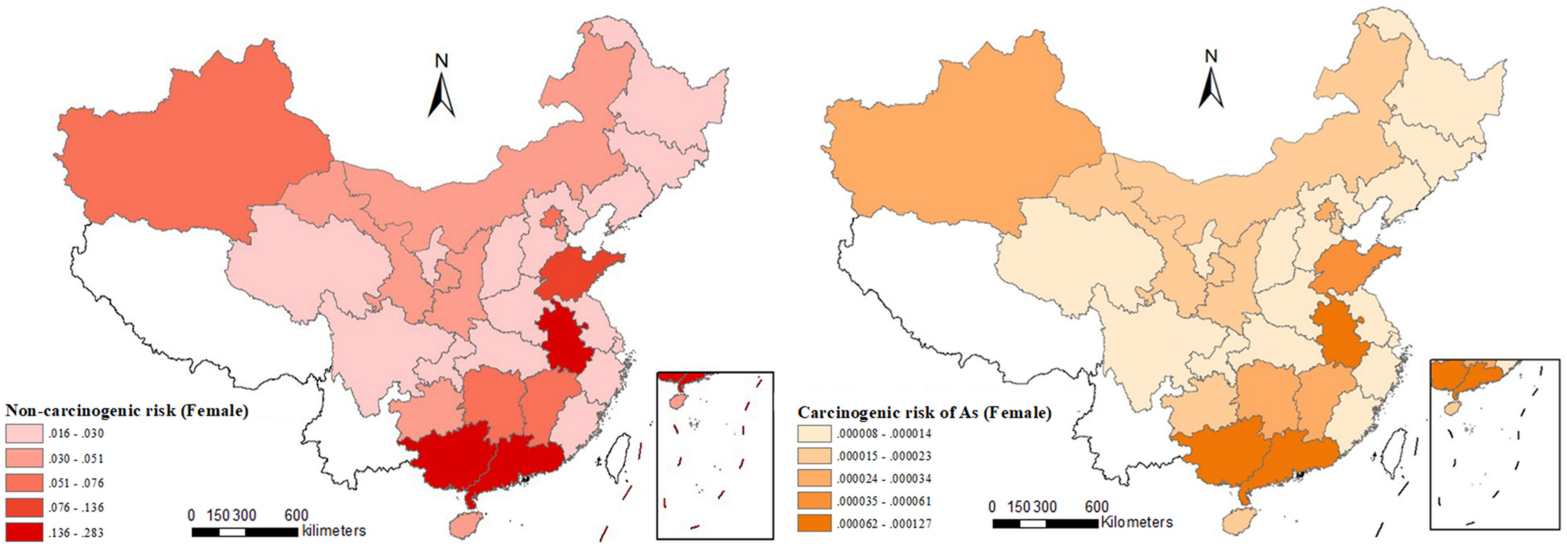
Health risk of As pollution for adult females in agricultural regions. Data source: Yang et al. (81).

In addition to heavy metal, some metalloid in the cropland, such as As, can cause serious health hazards. Acute and chronic As exposure would result in disorders of the cardiovascular and other system, which may ultimately lead to cancer (81). According to the estimation of Yang et al. (81), as shown in Figure 6, the health risk caused by As pollution in the soil is high in South China and predominantly distributed in Yunnan, Anhui, Hunan, Guangdong, and Guangxi.

### Water Pollution

Water contributes over 40% of agricultural production.^6^ As the second largest economy and most populous country in the world, per capita freshwater resources of China only stand at 2,100 m^3^, which is roughly 28% of the international average. This makes China one of the most water scarce countries in the world (82). In addition, the spatial distribution of water resources of China is very unbalanced. About 82% of the water resource is distributed in the south of Yangtze River, where only 38% of the cropland is located. The Yellow River basin, Huaihe River basin, and Haihe River basin in North China only occupy 6.6% of water resources of the country but have 40% of cropland of the whole country (83). What is worse, due to rapid and intensive urbanization and industrialization, as well as loose environmental regulation, the scarce water resource has become severely polluted (84, 85).

**Figure 7:**
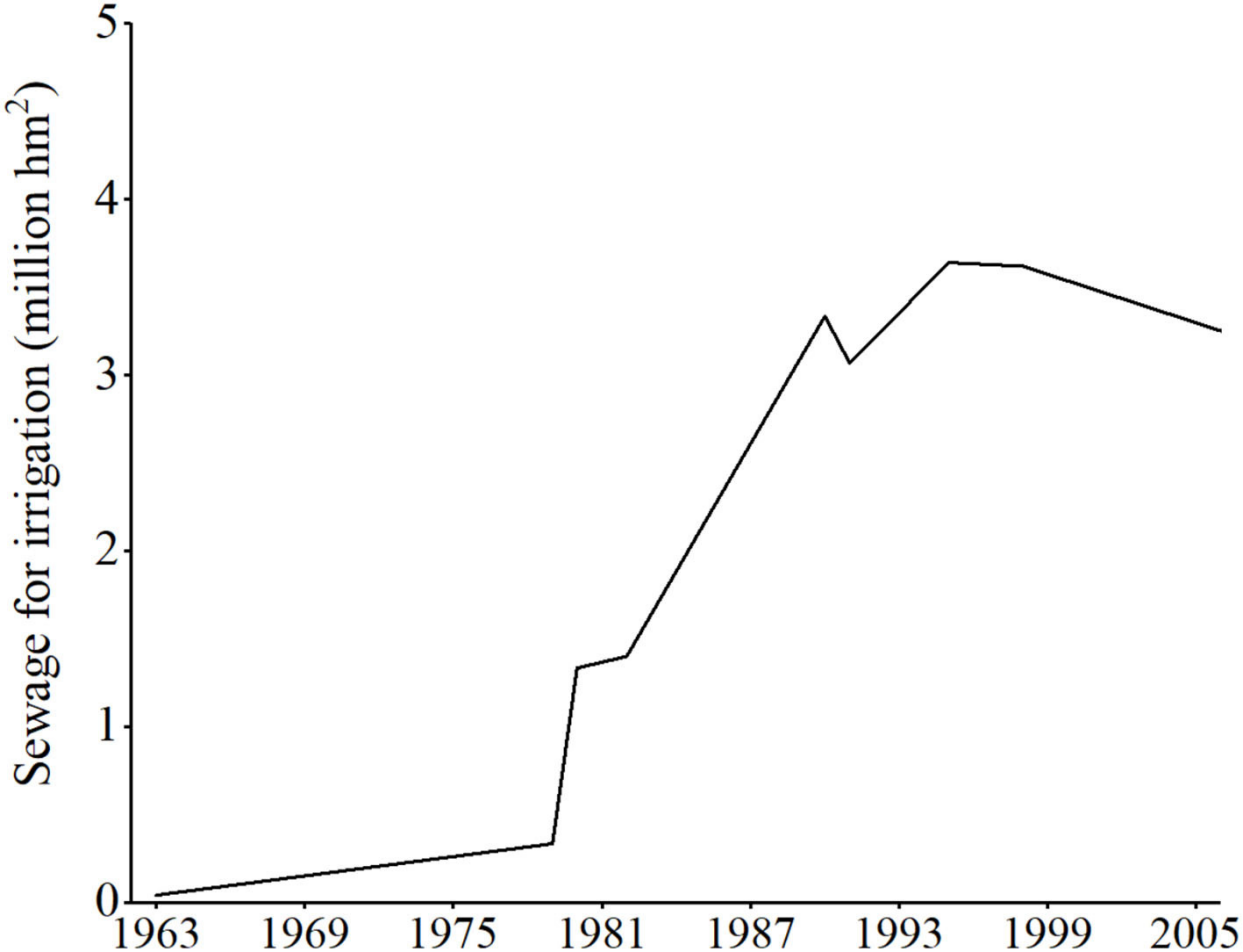
The amount of sewage for irrigation in China. Data source: National Agricultural Environmental Quality Survey Cooperation Group of Sewage Irrigation Area (86), Yang (87), Liu and Xu (88), and Liu et al. (89).

Since 1950's, China has used sewage for cropland irrigation (13), especially in the suburban areas of northern metropolises, such as Beijing, Tianjin, Shenyang, and so on. As shown in Figure 7, after 1970's, the amount of sewage for irrigation has soared. As of 1995, 3.62 million ha of cropland was irrigated by sewage, accounting for 7.33% of the total irrigated area in China, and 86% of the sewage was below the standard of irrigation water quality (90). As of 2006, there was still 216,666 ha of cropland irrigated by sewage.

Long-term use of sewage for irrigation will pollute cropland and agricultural products with excessive heavy metal and pathogens. Table 4 lists the heavy metal/metalloid concentration in some sewage irrigated areas. Accordingly, almost every surveyed area has pollutants exceeding the National Food Safety Standards (GB 2762-2012).

**Table 4:** The heavy metal/metalloid concentration in some sewage irrigated areas (mg/kg).

**Region**	**Grain**	**Cu (mg/kg)**	**Cd (mg/kg)**	**Cr (mg/kg)**	**Pb (mg/kg)**	**As (mg/kg)**	**References**
**Standard**		**10**	**0.2**	**1.0**	**0.2**	**0.15**	**GB 2762–2012**
Zhengzhou, Henan	Rice	-	0.016	0.21	**0.53**	0.10	(91)
	Wheat	-	0.02	0.18	**0.99**	0.11	
Baiyin, Gansu	Wheat	6.84	**0.61**	-	**1.29**	-	(92)
Beijing	Wheat	6.09	0.04	**4.62**	0.17	-	(93)
Anhui	Rice	8.7	**0.21**	-	0.06	**0.18**	(94)
Hebei	Wheat	1.79	0.015	0.69	0.15	-	(95)
Baiyin,Gansu	Wheat	7.61	**0.75**	-	**9.96**	-	(96)

*Pollutants exceeding the standard are marked bold*.

Table 5 lists the pathogen concentrations in some sewage irrigated areas. As shown in Table 5, the detection rates of intestinal pathogenic bacteria in the sewage irrigated areas in Beijing and Chifeng are, respectively, 27.9 and 25%, while the detection rates in the clean water-irrigated areas in two cities are only 2.65 and 8.39%.

**Table 5:** The pathogen content in some sewage irrigated areas.

**Region**	**Pathogen**	**Detection rate**	**Concentration**	**References**
Beijing	Intestinal pathogenic bacteria	27.9%		(97)
Chifeng, Inner Mongolia	Intestinal pathogenic bacteria	25%		(98)
Beijing	*Escherichia coli*		8.9 ± 0.19 × 10^4^ (CFU/mL)	(99)
Beijing	Fecal coliforms		2.4 × 10^5^ (CFU/mL)	(100)
Beijing	*Escherichia coli*		1.0 × 10^3^-1.1 × 10^4^ (CFU/mL)	
Shijiazhuang, Hebei	*Escherichia coli*		3 × 10^4^ (CFU/g)	(61)

According to the Ministry of Environmental Protection and Ministry of Land Resources (10), out of the 55 sewage irrigated areas investigated, 39 still have soil pollution. Therefore, although the Chinese government has prohibited the irrigation with sewage with heavy metals and/or persistent organic pollutants since 2013, the policy implementation is challenging, especially for the North China Plain, where the qualified water is severely lacked.

### Air Pollution

Air pollution, *inter alia*, ground-level O_3_ has been found to damage crops through leaf stomatal uptake, followed by a reaction with internal plant tissues to generate reactive oxygen species that interfere with various physiological functions (101). As shown in Table 6, the field experiment results in China also prove that O_3_ pollution significantly reduces crop yield. In the past decades, O_3_ concentration of China has been rising faster than in other countries, and the mean of the daily 24-h average concentration has reached over 50 ppb during the crop-growing season in some regions (105).

**Table 6:** The experiment results on the impact of O_3_ on crop yield.

**Site**	**Crop**	**Control**	**Treatment (O_**3**_)**	**Crop yield**	**References**
Jiaxing city, Zhejiang province	Wheat	Charcoal filtered air	75/100 ppb	↓ 8.5–58.0%	(102)
	Wheat	Charcoal filtered air	150/200 ppb	↓ 40–73%	
Jiangdu district, Jiangsu province	Winter Wheat	Non-filtered ambient air	25% above the ambient O_3_ concentration	↓ 20%	(103)
	Rice			↓ 12%	
Gucheng county, Hubei province	Wheat	Charcoal filtered air	Non-filtered ambient air	↓ 4.7%	(104)
			50 ppb	↓ 10.5%	
			100 ppb	↓ 58.6%	
	Rice	Charcoal filtered air	Non-filtered ambient air	↓ 7.3%	
			50 ppb	↓ 8.2%	
			100 ppb	↓ 26.1%	

Overall, Wang and Mauzerall (106) projected enormous reductions in yields of winter wheat (63%), summer corn (64%), and soybeans (45%) in China by 2020 based on SUM06 metric (sum of hourly concentrations of O_3_ above 60 nmol·mol^−1^). Tang et al. (107) estimated that, compared with 2000, under a high scenario of O_3_ concentration, the wheat yield would be reduced by 8.7% (10.2–11.5 mt) by 2020. Liu et al. (108) employed dose–response functions from China and estimated that the O_3_-induced-relative yield loss (RYL) for rice in Chongqing from 1990 to 1995 was 1.1–5.8% and would reach 10.8% in 2020, while, for winter wheat, it was estimated to be 0.2–9.8% in 1990 and would reach around 12% in 2020. In the Yangtze River Delta, RYL for rice was 2.5–6.6% from 1990 to 1999 and would reach 9.2% in 2020, while, for winter wheat, RYL was estimated to reach about 12% in 1999.

## The Policy Response of China to Fight Against the Pollution-Induced Food Safety Problem

In order to fight against the pollution-induced food safety threat, the Chinese government has made and adopted a large number of policies. However, there are no special decrees for the pollution-induced food safety issue. The relevant policies are all distributed in different legislations. Based on bibliometric and policy document analysis, this paper identifies 4,471 relevant articles in 1,180 policies and analyzes the policy evolution concerning the pollution-induced food safety issue of China.

### Data Source and Analyzing Methodology

#### Data Source

The primary source of the policy documents is pkulaw.com, which is an authoritative and comprehensive database documenting Chinese laws and regulations at different government levels since 1949. This study focuses on the policies at the national level, i.e., the policies issued by the central authorities, including the central government and national ministries.

To search for relevant policies, the first step is to determine the policy keyword. The pollution-induced food safety issue may be mentioned in both decrees on environmental protection and food safety. Therefore, from the China National Knowledge Infrastructure (CNKI) database, this study first obtained all the published literatures with “food safety” and “environmental pollution” in titles, abstracts, and keywords, and got 3,937 pieces of literature about food safety and 5,829 pieces of literature about environmental pollution. Next, since the keyword provides the most important information of literature, this study determined the policy keywords according to the high-frequency keywords in the pieces of literature and their co-words. For example, three of the high-frequency keywords were “Pharmaceutical sewage,” “Printing and dyeing sewage,” and “Aquaculture sewage.” “Sewage” was then selected as a policy keyword to involve the relevant policies as extensive as possible. Finally, this paper used “food,” “pesticide,” and “agricultural product” as the policy keywords for food safety, and “environment,” “green development,” “heavy metal,” “pollution,” and “sewage” as the policy keywords for environmental protection.

Next, this paper used the combination of one keyword for food safety and one keyword for environmental protection to conduct full text search in pkulaw.com and extracted the decrees where the two keywords were in the same articles. A total of 6,440 decrees were initially obtained. Furthermore, the authors read the title of each decree and excluded the irrelevant ones. After that, the authors extracted the policy articles containing both keywords for food safety and environmental protection and read the articles to identify their relevance. Finally, a database consisting of 4,471 articles on the pollution-induced food safety issue in 1,180 polices was built.

#### Analyzing Methodology

This study used a text mining technique to analyze the selected policy texts. Firstly, based on the Python Jieba module, the sentences in the text were separated into individual words to obtain the basic units for analysis. Since Python may separate a proper noun into multiple basic words, this study customized additional nouns for special organizations and regions in Python Jieba modules, such as “Standing Committee of the National People's Congress” and “Yangtze River Delta.” Second, based on the stop words list developed by Harbin University of Technology, the meaningless words, such as “overall” and “in short,” were removed. Third, the keywords in the documents were extracted with the Jieba module and the TF-IDF model. Based on the Bag-of-Words model, the TF-IDF model is used to evaluate the importance of a word in the text and extract the keywords based on unique representation instead of frequency (109).

In addition, this paper employed co-word analysis and convergence of iterated correlations (CONCOR) clustering to visualize the connection between the policy keywords (110). The co-occurrence of the extracted keywords was firstly counted to establish a co-word matrix, which was imported into UCINET software to generate a visualization network. Furthermore, CONCOR calculates the inclusion index of each pair of keywords to find out the interrelationships of these keywords, which is used to trace the policy focus of different periods (111).

### An Overview of the Relevant Policies

**Figure 8:**
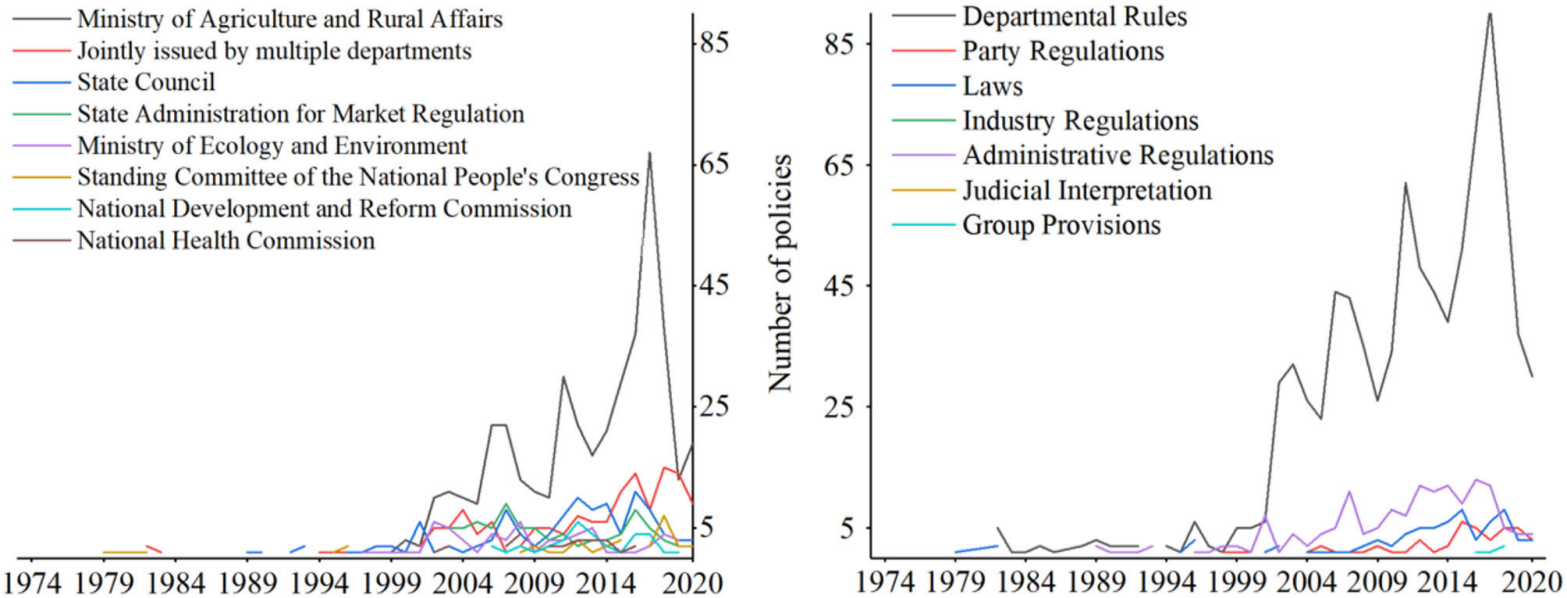
The number of policies involving the pollution-induced food safety issue.

As shown in Figure 8, generally, the number of policies involving the pollution-induced food safety issue has significantly increased with fluctuation since 1970's, with a peak in 2017. A notable increase was after 2006, when the “Law on the Safety of the Agricultural Product Quality” was implemented.

According to the classification of *pkulaw.com* on the legal status and authority of government decrees, Figure 8 counts the number of policies belonging to laws, departmental rules, party regulations, administrative regulations, industry regulations, judicial interpretations, and group provisions. Accordingly, the laws, departmental rules, and administrative regulations are the main policy categories. The law has the strongest legal force, and any other provision shall obey the law. Since the promulgation of the “Law on the Safety of the Agricultural Product Quality” in 2006, the number of relevant laws began to rise rapidly, providing legislative support for the control of the pollution-induced food safety problem, such as the “Administrative Measures for the Safety Management of Agricultural Producing Area” in 2006, and the “Measures for the Administration of Pesticide Labels and Manuals” in 2007. Second, to the laws, the administrative regulations made by the State Council play a critical role in providing guidance for specific actions. The number of department rules is the largest. The aim of department rules is mainly proposing feasible and specific measures to implement relevant central policies, so its fluctuation trend is very similar to the administrative regulations.

The central government and over 54 ministries have ever issued relevant policies. Among them, the State Council, National Development and Reform Commission, State Administration for Market Regulation, National Health Commission, Ministry of Agriculture and Rural Affairs, Standing Committee of National People's Congress, Ministry of Ecology and Environment are the main policymakers, having issued over 30 policies, respectively.

**Figure 9:**
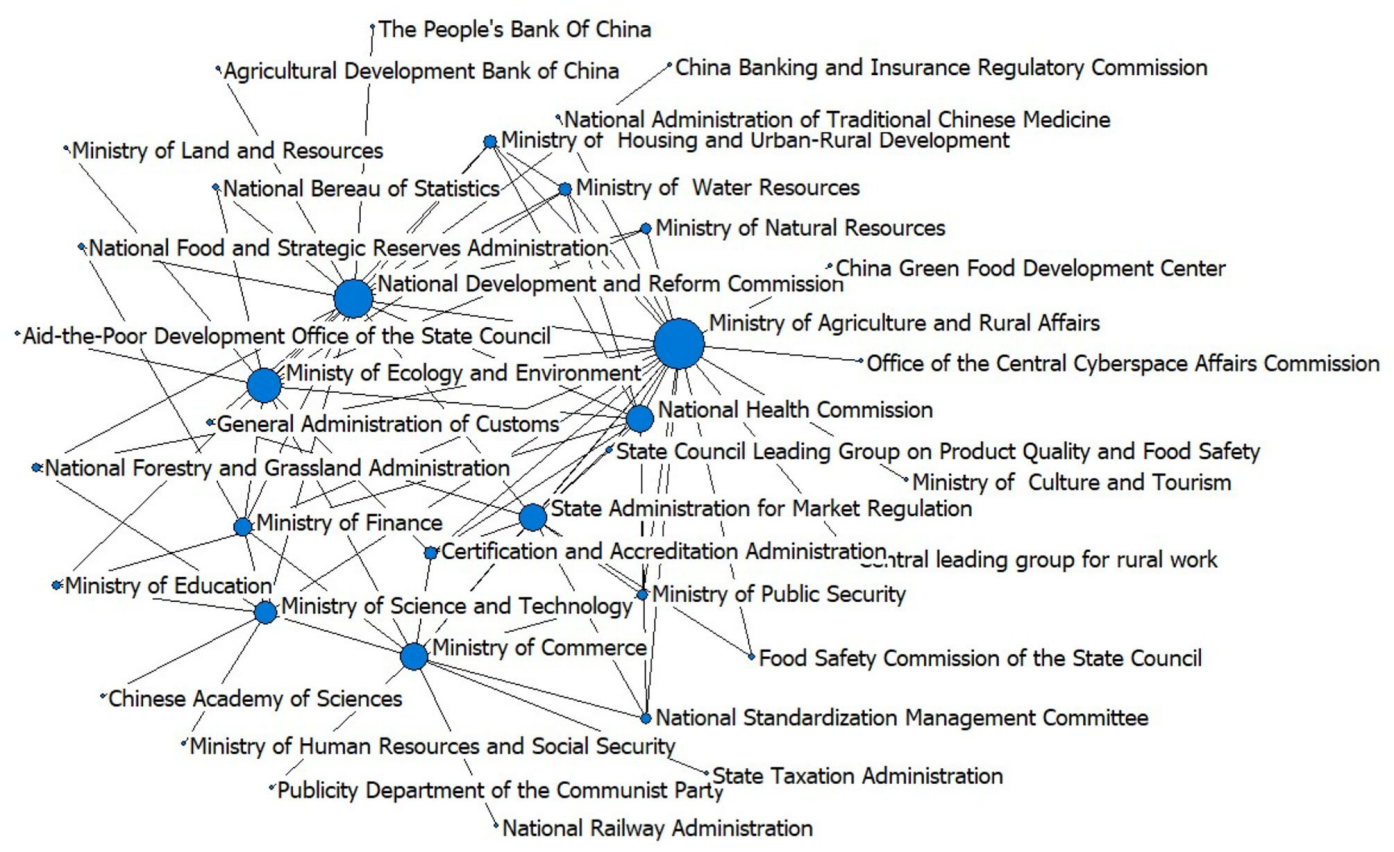
The cooperative network in jointly published policy documents.

Since the “Notice of the State Council on Strengthening the Work of ‘Vegetable Basket' in the New Stage” in 2002, the number of policies jointly issued by different departments has substantially increased. According to Figure 9, a cooperative network of 42 departments with the Ministry of Agriculture and Rural Affairs, National Development and Reform Commission and Ministry of Ecology and Environment as the main cores has been established. Since the organizational reform of the State Council in 2018, the functions of the ministries of food, agriculture, environment, and other related fields have been further sorted and integrated. The number of jointly published documents has thus decreased. However, currently, the problem of agricultural products is mainly the responsibility of the agricultural agency, while the environmental agency mainly works on the general pollution issues. So, the pollution-induced food safety problem still stands at the intersection of agricultural and environmental agencies, which may affect the efficiency and effectiveness of pointed policymaking.

### The Policy Characteristics in Different Periods

**Figure 10:**
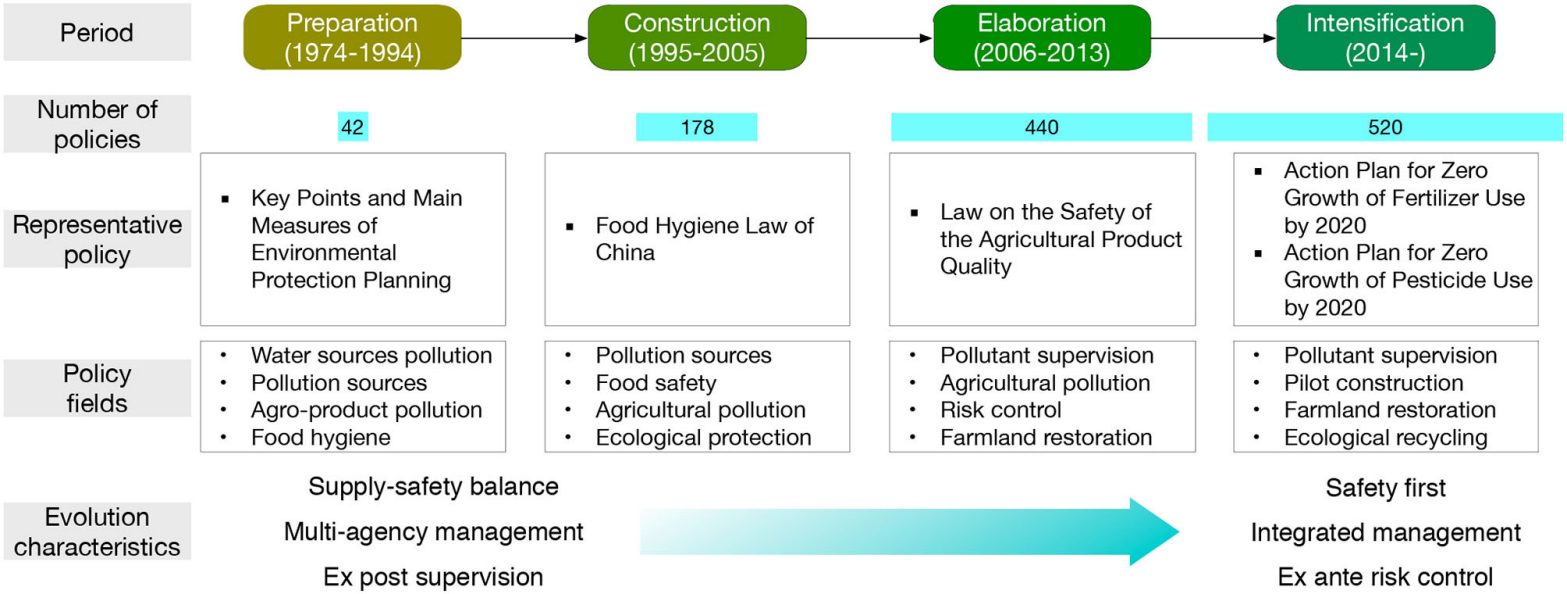
The policy evolution on pollution-induced food safety of China.

As shown in Figure 10, according to the time when landmark legislations were promulgated, this paper divides the policy evolution on pollution-induced food safety of China into four stages, i.e., preparation stage (1974–1994), construction stage (1995–2005), elaboration stage (2006–2013), and intensification stage (2014–) and analyzes the policy characteristics in different stages.

#### Preparation Period (1974–1994)

In 1974, the Chinese government started to pay attention to the pollution-induced food safety problem. For the first time, the Environmental Protection Leading Group of the State Council limited the pesticide residues in crops in the “Key Points and Main Measures of Environmental Protection Planning.” Up to 1994, 42 relevant policies had been issued, and most of them appeared in the relevant environmental policies proposed by the environmental agency.

**Figure 11:**
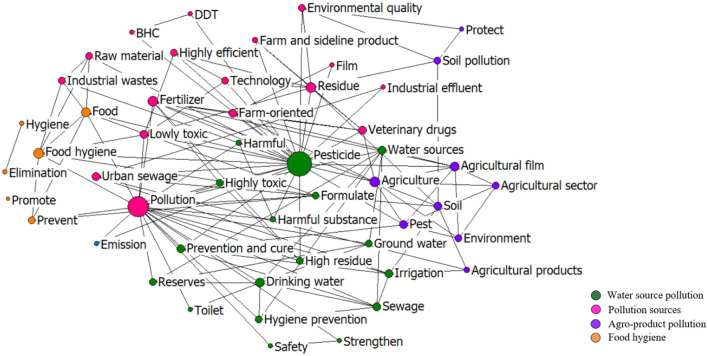
The co-word network of the keywords in the policies in 1974–1994.

Figure 11 shows the co-word network of the top 50 keywords in the policies implemented from 1974 to 1994. Accordingly, “pesticide” and “pollution” are the most highlighted nodes in this stage. All the keywords can be clustered into four categories: agro-product pollution, pollution sources, food hygiene, and water source pollution. Among them, water source pollution is the most important policy topic. With the implementation of reform and opening policy in 1978, the rural enterprise began to develop rapidly, and sewage irrigation entered a fast-developing period. Therefore, the Chinese government brought out and revised a series of irrigation water quality standards, which were applied to surface water, groundwater livestock and poultry breeding wastewater, wastewater from agricultural product processing, and rural domestic sewage in 1979, 1985, and 1992, respectively.^7^

Reflected by the keywords such as “prevent,” “formulate,” and “strengthen,” in this stage, the government mainly adopted directional measures regarding the restrictions of highly toxic pesticides and chemical fertilizers, such as the “Administrative Rules for Pesticide Registration” in 1982 and “Notice of the General Office of the State Council on Strengthening the Management of Pesticides and Veterinary Drugs” in 1992. High efficiency, safety, and economy were the main targets for the chemical input monitoring in this stage. The measures included, e.g., “improving the chemical fertilizer structure and fertilization technology,” “well-detecting the pesticide and veterinary drug residues in the domestic and exported agricultural products,” and “registering fertilizers and pesticides produced and sold by any organization and individual and involving the chemical residues and the impact on the atmosphere, water, and soil in the registration.”

However, in a country with such a large population, keeping the growth of grain output and ensuring a stable food supply had always been a requisite for China. Therefore, the government still had a strong dependence on chemical inputs to boost crop production. From the 6th Five-Year Plan (FYP) for National Economy and Social Development (1981–1985) to 8th FYP (1991–1995), increasing the supply of chemical fertilizers and pesticides was still listed as a main task.

#### Construction Period (1995–2005)

By the end of the twentieth century, the food supply in China was basically stable and sufficient. The government and society thus started to pay more attention to food quality and safety. In 1995, the first food law of China, “Food Hygiene Law of the People's Republic of China,” was promulgated, raising the pollution-induced food safety issue to the judicial level. From 1995 to 2005, a total of 178 policies were issued, establishing the solid institutional foundation for the regulative actions.

**Figure 12:**
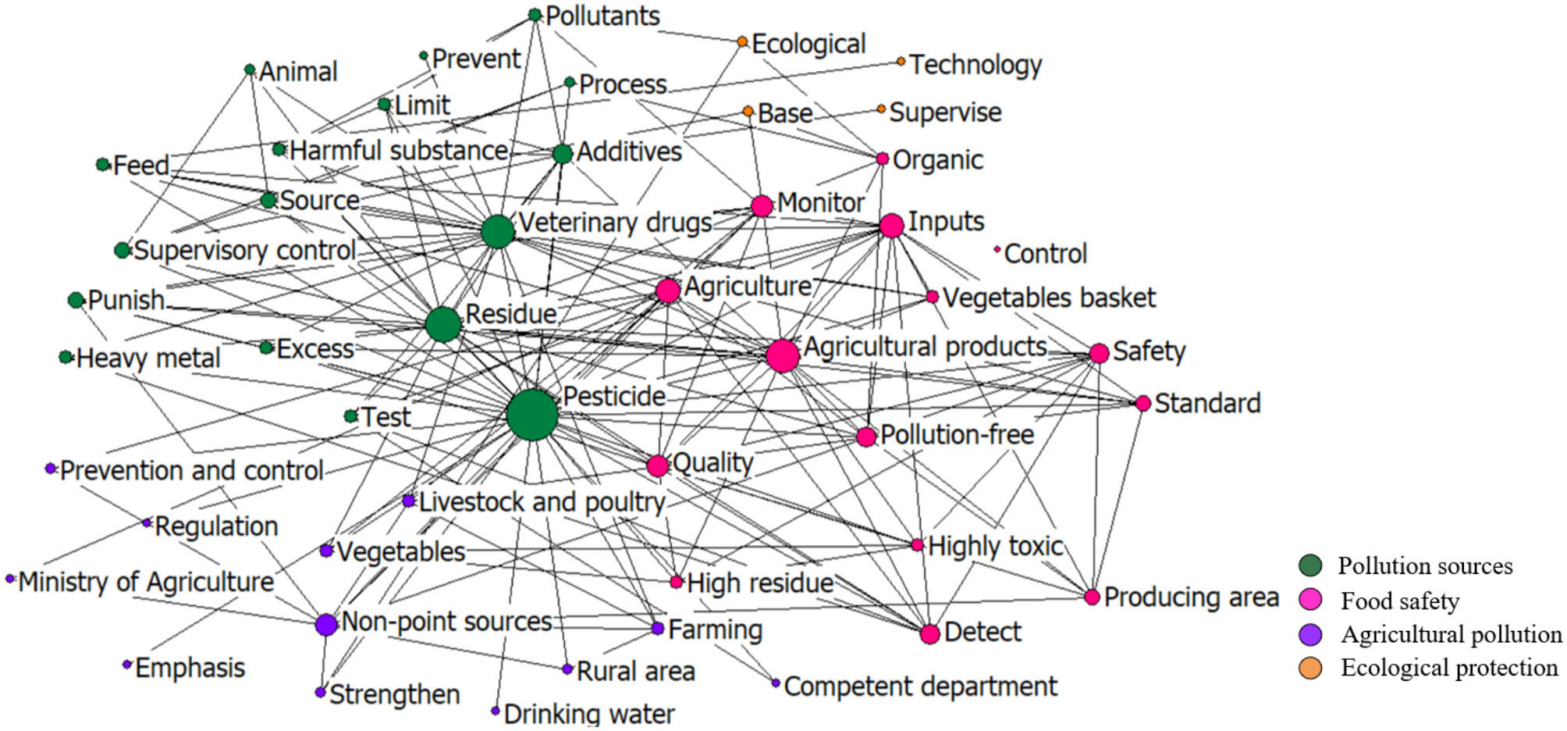
The co-word network of the keywords in the policies in 1995–2005.

According to the co-word network of the top 50 keywords during 1995–2005 (Figure 12), “pesticide” remained the main policy keyword. Some new keywords, such as “vegetables,” “feed,” and “growing area” emerged in this period, indicating that the government paid more attention to the pollution in agricultural production. The clusters of the keywords, including pollution sources, ecological protection, agricultural pollution, and food safety, indicate that the government still prioritize the monitoring of chemical inputs and residues, while, at the same time, began to keep eyes on the production of non-pollution food and environmental management of food-producing areas. In addition, the management measures in this period became more standardized and proactive, reflected by the keywords such as “prevention,” “standard,” and “plan.”

In this stage, with the increasing international requirements for the quality of exported agricultural products and improvement of awareness of the government on environmental protection, the focus of the chemical input supervision was shifted from supply to security, including low toxicity, low residue, and high efficiency. The target was to produce more “organic food,” “non-pollution food,” and “green food.” Various measures, such as standard management, market-access control, routine monitoring, follow-up supervision, and sampling inspection, were adopted. The policy innovation was primarily reflected in the “Management Measures of the Organic Food Certification” in 2001, and the “General Principles for the Examination of Food Quality Safety for Market Access” in 2004.

In addition, the food safety supervision became more precise. For example, in 2005, the “Hygienic Standard for Grains” (GB 2715-2005) divided the pollutant residues into that in vegetables, rice, soybean, tea, and other food crops. In this period, besides government regulation, more diverse measures and social power were involved to prevent and control the food pollution. For example, the China Rural Special Technology Association was established in 1995 to deliver better knowledge of the appropriate use of agrochemicals for farmers. With the strengthening of public consciousness on food safety, the central government began to regularly release the testing reports of chemical residues in the inspected food samples in order to meet the public right to know and further improve the quality supervision of the agricultural products.

#### Elaboration Period (2006–2013)

In this period, the environmental pollution-oriented food management system was initially formed. With increasing reports on the pollution of agricultural products, in 2006, the “Law on the Safety of the Agricultural Product Quality” was promulgated to reinforce the control of the pollution-induced food safety problem with the rule of law. Since 2006, the chemical fertilizer consumption intensity has gradually slowed down. From 2006 to 2014, a total of 440 decrees involving pollution control of agricultural production were issued.

**Figure 13:**
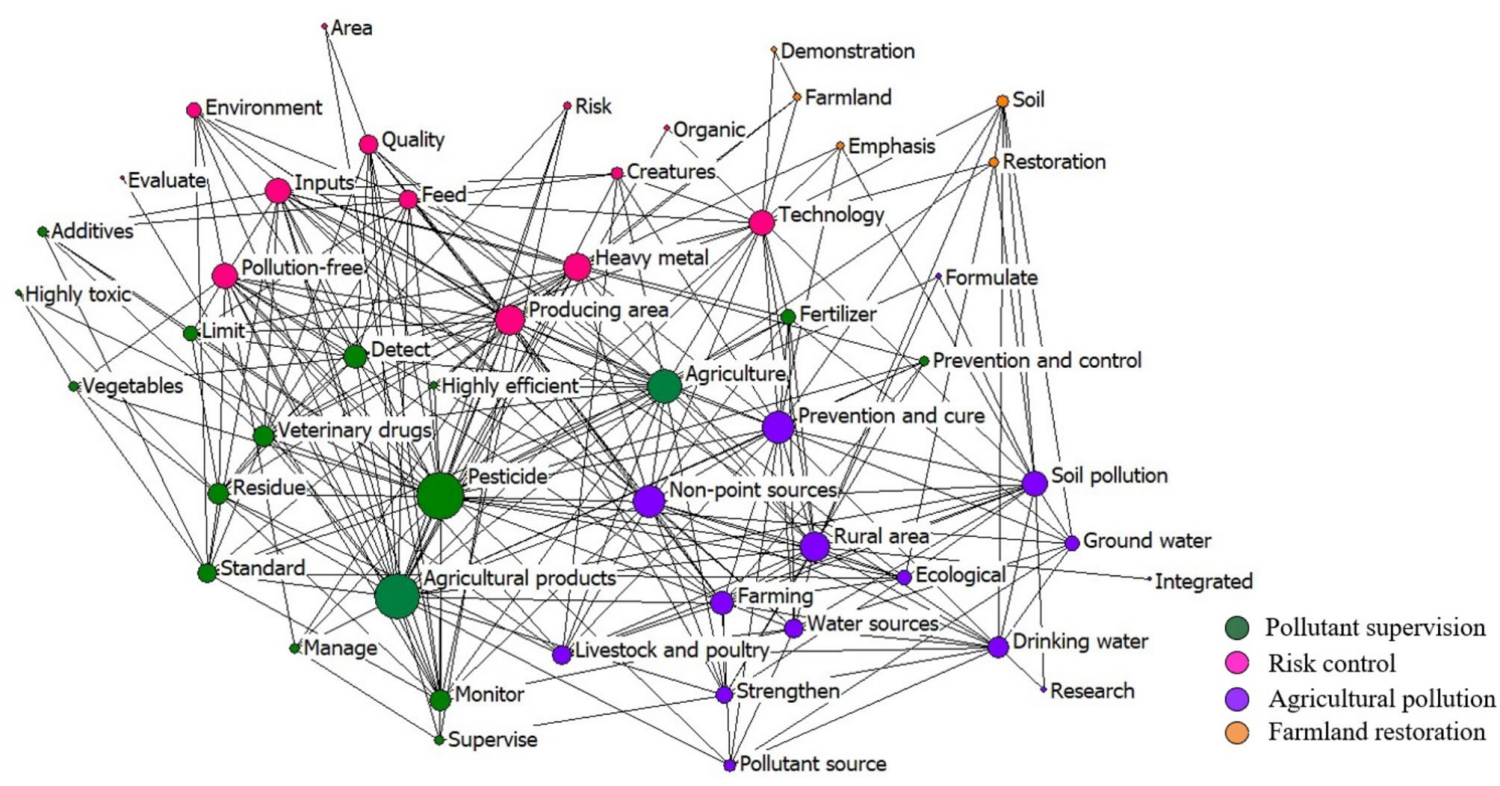
The co-word network of the keywords in the policies in 2006–2013.

Figure 13 shows the co-word network of the top 50 keywords in the policies implemented from 2006 to 2013. Accordingly, the supervision of chemical inputs remained the main policy keywords. However, the policy measures became more stringent, with the ban on the use of high-toxic pesticides. The clusters of the keywords show four policy arenas: pollutant supervision, risk control, agricultural pollution, and farmland restoration. With the frequent food pollution incidents such as the “turbot incident” in 2006,^8^ Cd poisoning in Zhuzhou in 2007^9^ and the Cd rice event in Hunan in 2013,^10^ the strategy of the government in this period gradually changed from *ex post* regulation to *ex ante* risk control.

First of all, more official standards were made to promote the standardized management of food pollution. For example, in 2007, China took the recommendation of the UN and started to implement the global standard on food safety management systems (ISO 22000). During 2011–2015, China formulated 4,140 pesticide residue limit standards and 1,584 veterinary drug residue limit standards (112).

In addition, the source control of food pollution was given more priority with a series of legislations and regulations issued to promote the pollution control in the place of origin of food, such as the “Administrative Measures for the Safety Management of Agricultural Producing Area” in 2006, “Measures for the Administration of Pesticide Labels and Manuals” in 2007, and “Inspection and Acceptance Method of Veterinary Drug Production Quality Management Standard” in 2010. In 2007, the National Agricultural Product Quality and Safety Risk Assessment Expert Committee was established to carry out risk analysis on the potential hazards that may affect the quality of agricultural products. In the same year, the Ministry of Agriculture at the time proposed to implement the coding of places of origin of agricultural products. By enabling the tracing of agricultural products to their original producing area, the regulatory authority is able to prompt the cleaner growing environment and production process.

In this period, the development of agricultural science and technology was also more emphasized. For example, the “Law on the Safety of the Agricultural Product Quality” in 2006 prescribed that the agricultural agency at the county level or above shall promote the construction of comprehensive demonstration areas for standardized agricultural production, demonstration farms, breeding areas, and delimited animal- and plant-disease free zones to explore the safe and pollution-free agricultural production and provide experiences for other regions. In the “Notice of Ministry of Agriculture on Distributing 2010 Financial Project Guide,” pesticide residue monitoring and agricultural product quality supervision were listed as the key research projects. In 2012, a monitoring network composed of one national, 31 provincial, 226 Prefectural and municipal, and 50 county-level monitoring institutions was established by the General Office of the State Council to monitor 154 indicators on pesticide residues, heavy metals, biological toxins, food additives, and food-borne pathogenic organisms, etc.

Moreover, to coordinate different regulatory bodies and improve the efficiency of legal enforcement, the central government established the State Council Leading Group on Product Quality and Food Safety in 2007. Following the promulgation of the “Food Safety Law” in 2009, the State Council Food Security Committee composed of several high-ranking officials was established to specify the roles of different ministries and administrations in food safety management. In 2013, at the first meeting of the 12th National People's Congress, the new National Food and Drug Administration was established and served as a centralized authority, replacing the functions of other regulatory bodies (113).

#### Intensification Period (2014–)

**Figure 14:**
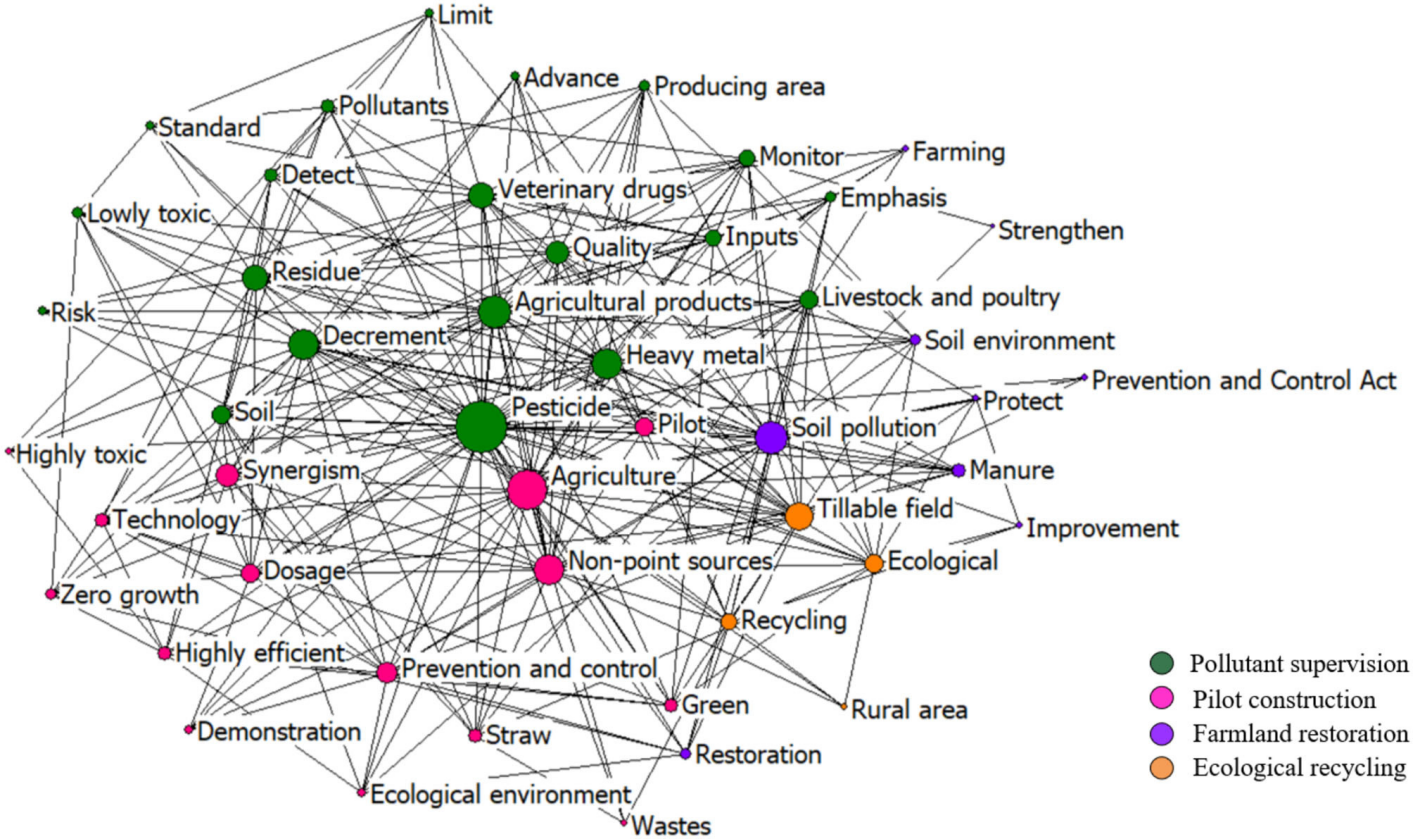
The co-word network of the keywords in the policies during 2014–2020.

With the promulgation of the “Action Plan for Zero Growth of Fertilizer Use by 2020” and “Action Plan for Zero Growth of Pesticide Use by 2020” in 2015, the strictest ever food pollution supervision system covering the whole food management process was set up, pushing the food pollution control into an intensification stage. According to Figure 14, after 2015, the chemical fertilizer and pesticide consumption in China started to reduce. So far, 520 relevant policies were issued in this period.

According to the co-word network of the top 50 keywords in Figure 14, the management of pesticides and other agricultural input remained the central policy keyword. The cluster of policy keywords gets four main policy topics: pollutant supervision, pilot construction, farmland restoration, and ecological recycling.

To intensify the quality control of agricultural products, in January 2014, the Ministry of Agriculture at the time announced 2014 as the “National Agricultural Product Quality and Safety Supervision Year.” Later, a series of important laws about pollution and food safety, including the “Environmental Protection Law of the People's Republic of China,” “Food Safety Law of the People's Republic of China,” and the “Law on the Safety of the Agricultural Product Quality” was successively amended in 2014, 2015, and 2018.

In this period, the pollution-control system in food management became more stringent with clear requirements for local governments and relevant stakeholders. For example, in 2016, “13th FYP for Hygiene and Health” of China (2016–2020) prescribed to formulate and amend no <300 standards on pesticides and veterinary drugs. The “Notice of the General Office of the State Council on the Key Work Arrangement of Food Safety in 2016” prescribed the accountability system of the provincial governors and the specific duties of each ministry on grain safety: the Ministry of Agriculture was mainly responsible for the environmental protection of the source of edible farm product, the Ministry of Environment was responsible for controlling heavy-metal pollution in grain, and the Ministry of Science and Technology and other relevant ministries were responsible for making standards for chemical residue.

Furthermore, strengthening the propaganda and education of legislations on the safety of agricultural products and agricultural inputs became an important measure to fight against the pollution-induced food safety problem. Besides, with the unprecedented emphasis on environmental protection in this period, the sustainable agriculture featured by ecology and environment-friendly and resource recycling is advocated and stressed. According to the “Guidelines for Eco Circular Agriculture Projects in Agricultural Comprehensive Development Areas (2017–2020)” issued in 2017, China would establish about 300 pilot programs for ecological and circular agriculture.

## Conclusion

Based on extensive literature study and policy document analysis, this paper reviews the environmental pollution-induced food safety problem in China, including the impact of environmental pollution on food safety and the policy response of the Chinese government since 1970's. The main findings of the research are as follows.

1. Agricultural development of China still relies heavily on agrochemical inputs, with the largest chemical fertilizer and pesticides consumption in the world, while the utilization efficiency is still much lower than that of the developed countries. Intensive but inefficient agrochemical consumption produces a large amount of residue in soil and plants and brings a great challenge to the food safety of the country.
2. The heavy metal pollution of cropland is salient that about 10.18% of cropland of China is polluted by heavy metals, and 13.86% of grain production is thus affected (11). Overall, the Cd pollution is the most prominent. The cropland heavy metal pollution in the central and southwestern China is relatively serious, represented by the provinces such as Hunan, Henan, Guangxi, Sichuan, and Chongqing. Gansu and Liaoning provinces in north China are also serious in heavy-metal pollution.
3. Water pollution, including general water pollution and sewage irrigation, is another important source for the food safety problem because the pollutants in the polluted water will be deposited in the cropland and transported to crops. Although the Chinese government has prohibited the irrigation with sewage with heavy metals and/or persistent organic pollutants since 2013, the policy implementation is challenging due to the lack of qualified water, especially for the North China Plain. According to the Ministry of Environmental Protection and Ministry of Land Resources (10), out of the 55 sewage irrigated areas investigated, 39 had soil pollution.
4. In the past decades, O_3_ concentration of China has been rising fast, and the mean of the daily 24-h average concentration has reached over 50 ppb during the crop-growing season in some regions (105). Current experimental and simulation studies have confirmed that a high concentration of O_3_ can largely reduce crop yield.
5. Since 1974, Chinese government has paid attention to the pollution-induced food safety problem. Up to 2020, the central government and over 54 ministries have issued about 1,180 policies involving 4,471 articles on the pollution-induced food safety issue. A cooperative network of 42 ministries with the Ministry of Agriculture and Rural Affairs, National Development and Reform Commission, and Ministry of Ecology and Environment as the main cores has been established. According to the time when landmark legislations were promulgated, the evolution of pollution-induced food safety policies of China can be divided into four stages, i.e., preparation stage (1974–1994), construction stage (1995–2005), elaboration stage (2006–2013), and intensification stage (2014–). Through the four stages, the supervision over the chemical input has always been the most important policy focus. The increasingly stringent policy system has been featured by “from supply-safety balance to safety first,” “from multiagency management to integrated management,” and “from *ex post* supervision to *ex ante* risk control.”

Last but not least, the pollution-induced food safety problem involves both agricultural and environmental agencies, so more collaborations between the two agencies and more special policies for pollution-induced food safety should be anticipated. In the meantime, more systematic studies over the causal relationship between environmental pollution and food quality, especially the emerging contaminants, such as microbial pathogens, persistent organic pollutants, and greenhouse gases, are also necessary for a profound understanding of the issue.

## Author Contributions

LL: designed the research. QL and KZ: collected data, conducted analysis, and wrote draft. LL and XS: edited the text and finalized the submission. All authors contributed to the article and approved the submitted version.

## Funding

The study was financially supported by the National Natural Science Foundation of China (Grant Number: 71704126).

## Conflict of Interest

The authors declare that the research was conducted in the absence of any commercial or financial relationships that could be construed as a potential conflict of interest.

## Publisher's Note

All claims expressed in this article are solely those of the authors and do not necessarily represent those of their affiliated organizations, or those of the publisher, the editors and the reviewers. Any product that may be evaluated in this article, or claim that may be made by its manufacturer, is not guaranteed or endorsed by the publisher.

## Footnotes

^1^FAO. Increased soil contamination puts food safety and food security at risk. http://www.fao.org/news/story/en/item/1173588/icode/ (accessed September 19, 2021).

^2^FAO. New UN Decade on Ecosystem Restoration offers unparalleled opportunity for job creation, food security and addressing climate change. http://www.fao.org/news/story/en/item/1182090/icode/ (accessed September 19, 2021).

^3^UNECE. Air pollution and food production. https://unece.org/air-pollution-and-food-production (accessed September 19, 2021).

^4^http://www.fao.org/faostat/en/#data/EF (accessed September 19, 2021).

^5^State Administration of Market Regulation. Circular of the State Administration of Market Regulation on the Food Safety Supervision and Sampling Inspection in the First Half of 2020. http://gkml.samr.gov.cn/nsjg/spcjs/202007/t20200730_320364.html (In Chinese) (accessed September 19, 2021).

^6^National food security strategy from the perspective of modern agricultural development. http://www.npc.gov.cn/npc/xinwen/2012-07/06/content_1729123.htm (In Chinese) (accessed September 19, 2021).

^7^The irrigation water quality has always been a policy focus that, after 1992, the standard was successively revised in 2005, 2008, and 2021.

^8^In 2006, Shanghai Food and Drug Administration detected nitrofuran metabolites in all the 30 aquatic product samples collected from a number of markets, and enrofloxacin, ciprofloxacin, chloramphenicol, malachite green, erythromycin, and other banned fish residues were detected in part of samples.

^9^In 2007, the crops and soil of Xinma village of Zhuzhou, Hunan was polluted by industrial pollution and high Cd-content fertilizer. Mild cadmium poisoning was found for 150 villagers.

^10^In 2013, Guangzhou Food and Drug Administration found over-standard Cd in eight of 18 batches of rice samples, and ^s^ix batches of them were produced in Hunan Province.
